# Feasibility and acceptability of home-based computerised cognitive training after cardiac surgery

**DOI:** 10.1186/s40814-026-01882-y

**Published:** 2026-07-16

**Authors:** Tracey Bowden, Julie Sanders, Catherine S. Hurt, Mandeep Sekhon, Leanne M. Aitken

**Affiliations:** 1 School of Health and Medical Sciences, City St George’s, University of London, London, EC1V 0HB UK; 2 St. Bartholomew’s Hospital, Barts Health NHS Trust, West Smithfield, London, UK; 3 Faculty of Nursing, Midwifery & Palliative Care, King’s College London, London, SE1 8WA UK; 4 Department of Population Health & Policy, School of Health and Medical Sciences, Tooting Campus, City St George’s, University of London, Cranmer Terrace, London, SW17 0RE UK

**Keywords:** Feasibility and acceptability study, Computerised cognitive training, Cardiac surgery, Postoperative cognitive decline

## Abstract

**Aims:**

Cognitive decline is common after cardiac surgery and is associated with poor postoperative outcomes. The aim of this study was to evaluate the feasibility and acceptability of a home-based computerised cognitive training programme to improve POCD following cardiac surgery; this study was not designed to assess the efficacy of the intervention.

**Methods:**

This single-arm, non-blinded, feasibility and acceptability study included patients ≥ 18 years undergoing first-time elective cardiac surgery using standard extracorporeal circulation. Participants were required to complete an 8-week computerised cognitive training (CCT) programme (20 min/day; 5 days/week), commencing 1 week after surgery. The Montreal Cognitive Assessment (MoCA) was administered preoperatively and after programme completion. Feasibility outcomes included recruitment and retention rates and programme adherence. Acceptability was assessed by the Theoretical Framework of Acceptability Questionnaire (TFA-Q).

**Results:**

Twenty-nine participants were recruited: 7 (24%) were female and 20 (69%) were White British. Study recruitment was deemed to be feasible (61% of eligible patients) and the CCT was acceptable to 94% of participants who completed the TFA-Q (*n* = 16), noting that this does not reflect the full recruited cohort. Intervention adherence (*n* = 9, 31%) and study retention (*n* = 17, 59%) were limited.

**Conclusions:**

The findings of this feasibility study suggest that home-based CCT is deliverable and acceptable to patients following cardiac surgery, though strategies to improve retention and adherence will need to be strengthened before progression to an efficacy study.

**Trial registration:**

NCT05298540.

**Supplementary Information:**

The online version contains supplementary material available at https://doi.org/10.1186/s40814-026-01882-y.

## Key messages

- Prior to this study, it was uncertain whether cardiac surgery patients would be willing and able to engage with a home-based computerised cognitive training programme during early postoperative recovery, and whether recruitment, retention, and adherence rates would be sufficient to support a full-scale trial.
- The study recruited 29 participants (61% of eligible patients) and achieved 59% retention at programme completion. Acceptability was high among those who completed the programme and returned the TFA-Q (*n* = 16 of 17, 94%). Overall intervention adherence was limited (31%), indicating challenges with programme completion during the early postoperative recovery period.
- The amber feasibility criterion was satisfied, supporting progression to a larger trial with design modifications including: enhanced recruitment strategies to improve patient identification and enrolment, revised intervention dose and timing to better accommodate postoperative recovery demands, refinements to the training protocol to optimise adherence, and consideration of a multi-centre approach to achieve adequate sample size.

## Introduction

Cognitive decline is common after cardiac surgery affecting between 30 and 80% of patients in the weeks following surgery, reducing to 10% to 60% after 3–6 months [1]. Standardised terminology now exists to describe postoperative neurocognitive disorders based on the timing of cognitive assessment [2]: deficits detected within 30 days of surgery are classified as delayed neurocognitive recovery, while those identified between 30 days and 12 months after surgery are termed postoperative neurocognitive disorder (POCD). Operationally, POCD is defined as a decline in cognitive performance from baseline to postoperative assessment, typically identified when a patient’s change in test scores exceeds a statically defined threshold [3], most commonly affecting memory, attention, psychomotor speed, and visuospatial ability [4, 5]. POCD is associated with poor postoperative outcomes, including prolonged hospitalisation [5], delayed return to work or loss of employment [6], reduced quality of life [7], and increased mortality [7], representing a significant burden on the health care systems [8].

Numerous pharmacological [9] and operative strategies [10] have been explored in an attempt to prevent POCD; however, limited clinically important improvements have been identified to date. Amongst non-pharmacological approaches, computerised cognitive training (CCT) is particularly promising as it offers a structured, scalable, and individually adaptive method of targeting multiple cognitive domains simultaneously, with emerging evidence suggesting benefit following cardiac surgery [11, 12]. Home-based delivery is especially relevant in the early postoperative period, when patients are recovering at home and face practical barriers to attending centre-based rehabilitation, and when early cognitive engagement may be beneficial [11]. Beyond logistical convenience, a home-based model also broadens equity of access, enabling participation among those who may face additional structural barriers to centre-based provision, including individuals with disabilities, caring responsibilities, or limited access to transport. Although few studies have examined some elements of using a cognitive intervention to support recovery after cardiac surgery [12, 13], feasibility data in UK populations are lacking, and no previous study has assessed acceptability using a theory-based framework. Thus, we sought to evaluate the feasibility and acceptability of a home-based computerised cognitive training (CCT) programme in the postoperative cardiac surgical population in the UK context.

## Patients and methods

### Ethical statement

The study was approved by the Research Ethics Committee and Health Research Authority (08/07/2022, Ref.: 22/WM/0112) and complies with the Declaration of Helsinki. Written informed consent of each patient was obtained at enrolment.

### Study design and population

A single-arm, non-blinded, feasibility and acceptability study was conducted at a tertiary referral hospital within one of the largest cardiovascular centres in Europe. This study was not designed to evaluate the efficacy of the intervention. The study reporting is consistent with the CONSORT extension for Pilot and Feasibility Trials Checklist [14]. A completed CONSORT checklist is provided in the supplementary information.

Adult patients (≥ 18 years old) undergoing first-time elective cardiac surgery with standard extracorporeal circulation, willing to engage with online cognitive training, with access to a computer or tablet with an internet connection were eligible to participate. Eligible patients were systematically identified by the cardiac clinical care team through the preoperative outpatient clinic or cardiothoracic wards, ensuring all eligible patients, including interhospital transfers, were invited to participate. Initial approach was made by either an advanced nurse practitioner or ward nurse in charge, with interested patients subsequently approached by the researcher in person or via post. All potential participants received a participant information sheet and informed consent form, and given at least 24 h to consider participation, and provided written consent prior to enrolment. Patients unwilling or unable to give written informed consent, those with significant psychiatric or medical comorbidities where that condition might impact cognitive function, an inability to understand written and/or verbal English, and those with motor symptoms that would impede their ability to complete the programme were excluded. It is acknowledged that the requirement for digital literacy and internet access may have introduced selection bias, potentially limiting generalisability of findings.

### Sample size

As a power calculation is not necessary for feasibility and acceptability studies, a sample of 30 patients was deemed sufficient to evaluate feasibility goals [15, 16]. The sample size calculation for a subsequent efficacy study (randomised controlled trial [RCT]) was calculated using the G*Power software [17] using a significance level [α] = 0.05, power 0.8, two-tailed probability tests, and an effect size Hedges’ g = 0.31. The effect size was based on a published meta-analysis [18].

### Intervention

BrainHQ (PositScience, San Francisco) a CCT intervention, used previously in surgical studies [19, 20] is a web-based and/or mobile software application designed to improve performance of specific cognitive abilities [21]. The exercises are based on the underlying principles of neuroplasticity [21] and targeted memory [19], attention [22], and processing speed [23] (Supplementary Table S1). The exercises were adaptive, meaning the difficulty levels were automatically adjusted to match the participants’ performance [21].

Participants were asked to complete 40 sessions (20 min/day, 5 days/week for 8 weeks) of CCT. This dose was selected to meet the BrainHQ recommended minimum training time of 600 min [21] whilst remaining realistic and achievable during the early postoperative period, when fatigue and competing demands on patients’ time are recognised challenges. Training commenced 1 week postoperatively to avoid the acute effects of sedatives, postoperative pain, sleep deprivation, and patient fatigue in an attempt to improve adherence [24]. Based on the schedule, up to 800 min of training and 320 levels (each level taking approximately 2.5 min to complete) could be achieved by the end of the intervention.

### Study procedures

**Fig. 1:**
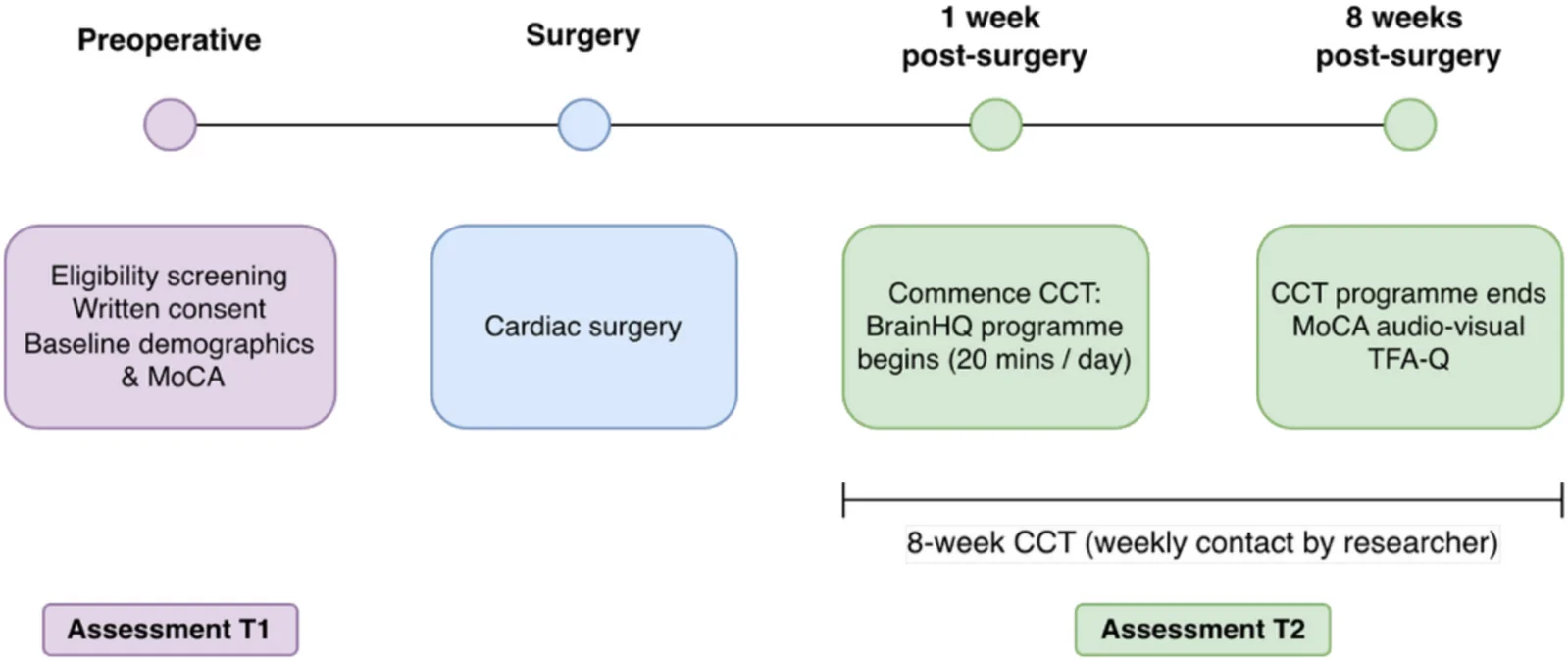
Study procedure and timeline of events

The study procedure and timeline of events are displayed in Fig. 1. Cognition was assessed by the Montreal Cognitive Assessment (MoCA) [25] at the first assessment (T1) and by the MoCA audio-visual at the second assessment (T2) by a study team member trained in MoCA administration. The MoCA is a cognitive screening tool and is not sufficient for formal diagnosis of POCD; within this feasibility study it was used descriptively to characterise the population at baseline and to note any changes following the intervention, rather than to assess efficacy. Outcome assessors were not blinded to participant progress, as assessments were conducted by the same researcher responsible for delivering the intervention support creating a potential source of bias. Before hospital discharge, participants were provided with written instructions on how to complete the CCT and intervention tracking logs at home. Participants were contacted weekly by the research team to address any technical issues and to encourage engagement with the CCT. Equipment required for the administration of the MoCA audio-visual at T2 (paper, pencil, eraser) was provided. After T2, details relating to medical history, preoperative risk factors, operative details, and postoperative outcome data were obtained from the locally held National Institute for Cardiovascular Outcomes Research (NICOR) National Adult Cardiac Surgery Audit (NACSA) and the Intensive Care National Audit and Research Centre (ICNARC) databases. Automated gameplay data, including total time trained (in minutes) and the number of levels completed, was downloaded directly from BrainHQ.

### Feasibility

Feasibility indicators were pre-defined with specific thresholds: recruitment success was defined as enrolling ≥ 50% of eligible patients [26]; retention was set at 75% of study participants completing the training and final assessment [27]; and intervention adherence was established as 75% of the programme with a minimum total training time of 600 min [21]. Additionally, the feasibility of testing procedures and data collection methods were evaluated by the researcher and participants by considering their practicality, resources needed, and potential impacts on study outcomes. Participant feedback regarding data collection procedures, where provided, including expressions of satisfaction or suggestions for improvement, were systematically documented.

### Acceptability

Acceptability of the intervention was assessed through the TFA-Q(28). This is a Likert-style questionnaire designed to evaluate participants understanding of content and relevance of an intervention. The questionnaire was adapted to the postoperative cardiac surgical cohort in collaboration with one of the developers of the TFA. The final questionnaire included questions related to 6 of the 7 TFA-constructs (affective attitude, burden, perceived effectiveness, intervention coherence, opportunity costs, and self-efficacy) as well as a general acceptability question. The ethicality construct was excluded on the basis that this construct relates to whether an intervention conflicts with an individual’s moral or ethical values, which was considered not applicable in the context of a cognitive training programme delivered to a cardiac surgical population [28].

### Statistical analyses

Descriptive statistics were used to characterise the demographic and clinical variables. Categorical data were summarised as frequencies and proportions. Continuous data were assessed for distribution (skewness and kurtosis) using the Shapiro–Wilk test [29]. Depending on the distribution of the data, continuous data are presented as mean and standard deviation (SD) or median and interquartile range (IQR). Descriptive statistics were used to explore the feasibility (recruitment and retention rates, time required to recruit to target, number of eligible patients required to recruit the target sample size, and adherence to the intervention) and acceptability of the home-based CCT. Using the general acceptability item, a single acceptability score (TFA-Q) was generated whereby scores of 4 or 5 were considered to be acceptable [28].

Post-hoc analyses compared (a) participants who completed versus did not complete the 8-week CCT programme, and (b) among completers, those who met versus did not meet the minimum 600 min target. These analyses were exploratory in nature and the study was not powered to detect statistically significant differences between groups. Group differences were analysed using independent t-tests, Mann–Whitney U tests, and chi-square tests (χ2) as appropriate for data distribution and type. Variables examined included age, sex, ethnicity, educational attainment, employment status, baseline MoCA, and EuroSCORE II. Bonferroni correction was applied to all post-hoc comparisons to control for Type 1 errors, with analyses performed using IBM SPSS Statistics version 28.

### Patient and public involvement and engagement

Two public and patient involvement and engagement (PPIE) contributors (a cardiac surgery patient and a carer) participated in the study design, management, conduct, and dissemination strategy development [30].

### Progression criteria

Proceeding to a larger efficacy study will be determined using a traffic light system applied to feasibility and acceptability indicators alongside calculated sample size requirements. This system categorises outcomes as: stop (red) for irremediable issues that preclude trial continuation; amend (amber) for addressable concerns requiring protocol modifications before cautious progression; or continue (green) for acceptable parameters supporting progression to the larger study [31].

## Results

### Participants

**Fig. 2:**
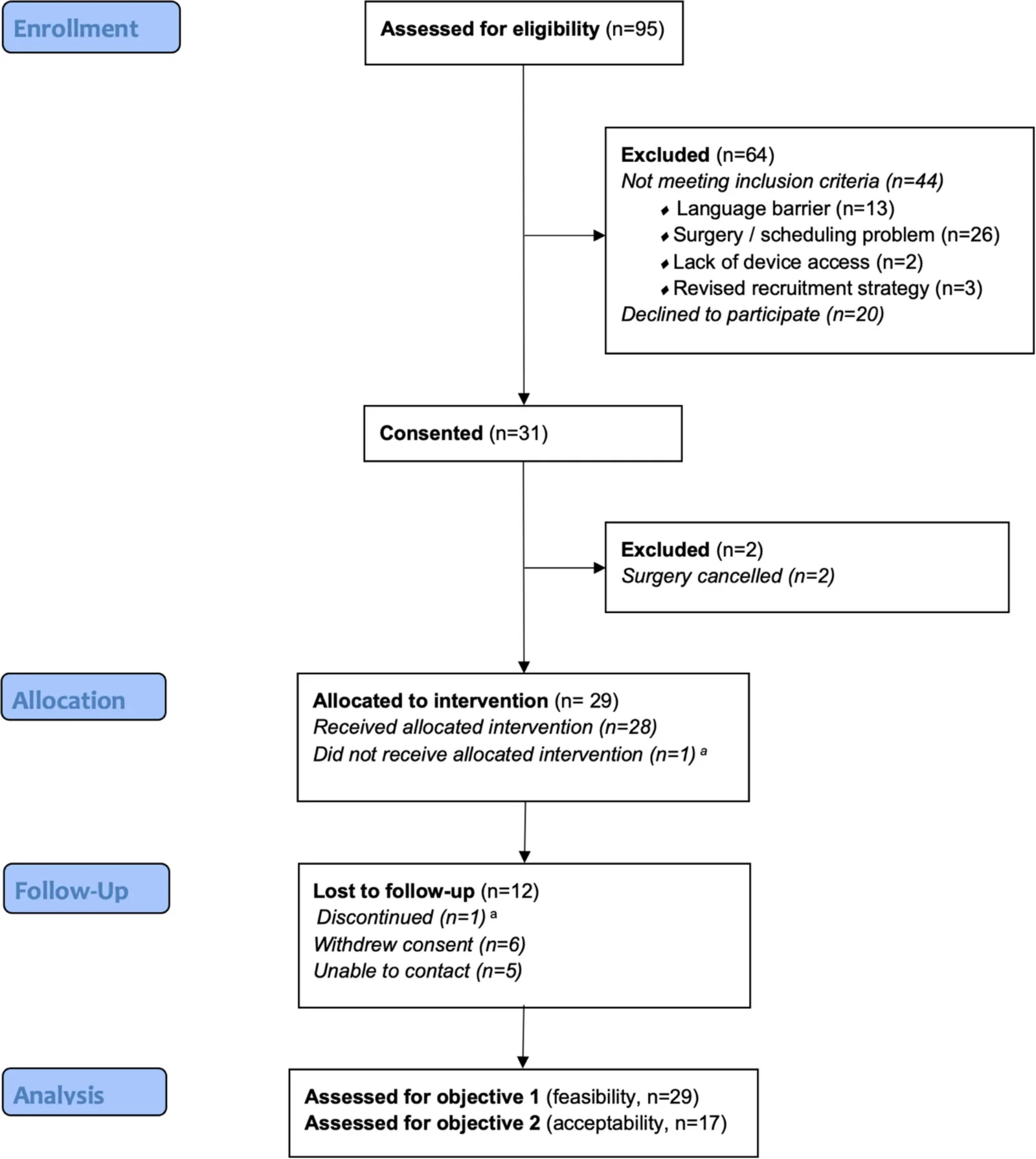
CONSORT flow diagram of study participants Note. ^a^ One patient was discontinued due to an intraoperative complication resulting in prolonged ICU length of stay and prolonged hospitalisation

Patients were enrolled between 1st August 2022 and 22nd September 2022 with the last follow-up completed on December 22, 2022. A total of 157 patients underwent first-time elective cardiac surgery during the study period; 95 (61%) were screened, 51 (54%) of those screened met the eligibility criteria, 31 (61%) consented to participate, and 20 (39%) declined to participate (Fig. 2). Most participants who declined did not provide a specific reason (*n* = 18, 90%), while two (10%) cited anxiety about their upcoming surgery. Of the 31 participants who consented to participate, two (6%) were later excluded due to cancellation of surgery. The final study cohort comprised 29 participants with a median age of 63.0 years (IQR 13.00), of whom seven (24%) were female. Most participants identified as White (*n* = 21, 73%), with representation from Asian (*n* = 4, 14%), Black (*n* = 3, 10%), and other ethnic backgrounds (*n* = 1, 3%). Educational attainment was distributed across technical/vocational qualifications (*n* = 9, 31%), high school/secondary education (*n* = 8, 28%), bachelor’s degrees (*n* = 5, 17%), and postgraduate degrees (*n* = 7, 24%). Participants demonstrated low surgical risk profiles with a median EuroSCORE II of 1.23 (IQR 1.20) and normal baseline cognitive function as indicated by a median MoCA score of 27 (IQR 2.80). Further participant characteristics are presented in Table 1 and Supplementary Table S2. Seventeen participants (59%) completed the T2 assessment and the TFA-Q.

**Table 1:** Participant characteristics (*n* = 29). All values n (%) unless otherwise stated

Demographic and risk factor variables	*n* (%)
Sex:	
Female	7 (24)
*Age, (years) median (IQR)*	63.0 (13.00)
Ethnicity	
White	21 (73)
Asian	4 (14)
Black	3 (10)
Other	1 (3)
Educational attainment	
Postgraduate	7 (24)
Bachelor’s degree	5 (17)
High school/secondary	8 (28)
Technical/vocational	9 (31)
EuroSCORE II, median (IQR)	1.23 (1.20)
Baseline MoCA, median (IQR)	27 (2.80)
Operative variables	
Type of surgery	
CABG	11 (38)
Valve	5 (17)
Major aortic	11 (38)
Other	2 (7)
Duration of surgery (minutes), median (IQR)	264.0 (90.30)
Bypass time (minutes), mean (SD)	104.8 (58.72)
Aortic cross-clamp time (minutes), mean (SD)	86.5 (51.67)
Immediate postoperative variables	
Return to theatre	0 (0)
Deep sternal wound infection	0 (0)
New postoperative neurological dysfunction	1 (3)
ICU length of stay (days), median (IQR)	4.1 (2.87)
Postoperative length of stay (days), median (IQR)	8.0 (7.50)
Total length of stay (days), median (IQR)	10.5 (8.00)

*Abbreviations*: *CABG* coronary artery bypass graft, *ICU* intensive care unit, *IQR* interquartile range, *MoCA* Montreal Cognitive Assessment, *SD* standard deviation

### Feasibility

**Fig. 3:**
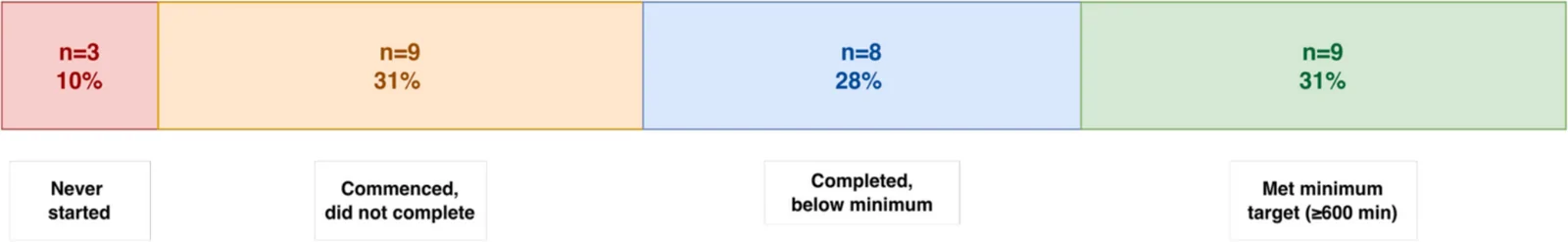
Participant engagement with the CCT programme (*n* = 29)

Thirty-one eligible patients (61%) consented to participate, meeting the predefined recruitment target. However, retention (*n* = 17, 59%) and adherence (*n* = 9, 31%) rates fell below targets. Engagement with the CCT programme varied across participants. Of the 29 participants, three (10%) never engaged with the CCT programme, 17 (59%) completed the 8-week programme and T2 assessment, and nine (31%) of the total cohort achieved the minimum 600 min of training (Fig. 3 and Supplementary Fig. S1). All participants meeting minimum targets completed the 8-week programme and final follow-up.

Of the 17 participants who completed the 8-week intervention, nine (53%) achieved the minimum 600 min of training, with seven reaching the target within 18–35 days. Only five participants (29%) commenced training on the planned date (1-week post-surgery) with delays ranging from 1 day (*n* = 3) and 2 days (*n* = 1) to 6–20 days (*n* = 8). No training patterns were observed among the 11 participants lost to follow-up.

### Acceptability

**Fig. 4:**
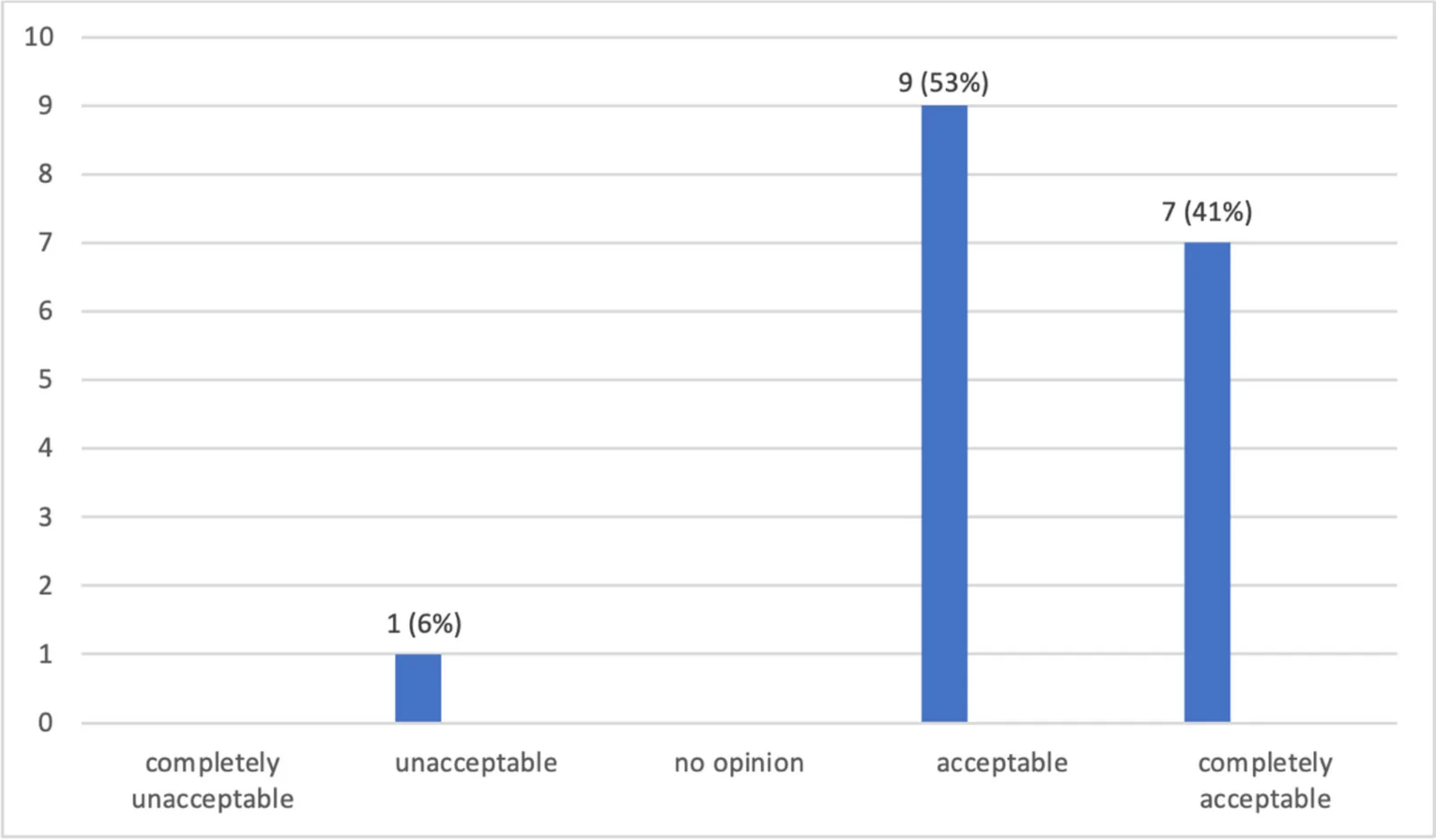
Acceptability of the CCT *Note*. Responses from participants who completed the 8-week intervention and returned the TFA-Q, representing 59% of the total recruited cohort (*n* = 29). Participants were asked ‘How acceptable was the home-based computerised cognitive training to you?’ (TFA-Q General Acceptability Item question 7)

Seventeen participants (59%) completed the TFA-Q. Responses to individual TFA-Q items and the general acceptability item are displayed in Fig. 4 and Supplementary Fig. S4, respectively. Of the 17 participants who completed the TFA-Q, most participants (*n* = 16, 94%) rated the intervention as ‘acceptable’ or ‘completely’ acceptable. Thus, the predefined acceptability target was met. One participant of the 17 (6%) did not rate the intervention as acceptable describing it as ‘repetitive’ and ‘boring’.

### Post hoc analyses

Post hoc analyses comparing characteristics of those participants who completed the 8-week CCT programme to those who did not show characteristics were comparable between the two groups. However, the duration of surgery was higher for participants who did not complete the protocol (Med = 287.0 min, IQR = 60.0 vs Med = 215.0, IQR = 87.5). No variables differed between participants who met the minimum target of 600 min and those who did not (Supplementary Tables S3–S7). These analyses were exploratory and the study was not powered to detect statistically significant differences.

### Sample size for a larger efficacy study

The total sample size required for a two-armed RCT is 328 participants (165 per group). Accounting for a participant attrition rate of 40% during the follow-up period, a sample size of 460 (230 per group) will be required.

### Feasibility outcome assessment

Following review of the feasibility outcomes by the FACCT study steering committee, there was consensus that the results satisfied the predefined ‘amber’ criterion. While not all feasibility indicators were fully met, the committee concluded that progression to a larger-scale randomised controlled trial is warranted with appropriate design modifications to address the identified barriers to recruitment and optimise programme adherence.

## Discussion

The aim of this study was to evaluate the feasibility and acceptability of a home-based CCT programme in the postoperative cardiac surgical population, for the first time in the UK.

The primary objective of this feasibility and acceptability study, to evaluate whether a full-scale trial of home-based CCT following cardiac surgery is warranted, was achieved. Following review by the FACCT study steering committee, there was consensus that the results satisfied the predefined ‘amber’ criterion, indicating that progression to a later study is appropriate with specific design modifications. Whilst recruitment was successful, retention and adherence fell below predefined targets and will require targeted strategies before proceeding to a larger efficacy study.

Findings indicated that home-based CCT was feasible in terms of recruitment rate, exceeding the predefined target of 50% of eligible participants and reaching the recruitment goal more rapidly than anticipated. The screening rate (61%) during recruitment compares favourably to similar studies in cardiac surgical populations [26], with refusal rates (20%) substantially lower than reported averages for interventional studies [32]. This suggests considerable patient interest in CCT within the population, with participants citing reasons including providing structure to their day, regular participant follow-up, and the perception that training sounded enjoyable. However, recruitment challenges highlighted important considerations for future research. Staff availability and timing issues affected patient identification, particularly impacting surgical outliers (surgical patients placed on non-surgical wards due to bed shortages) who were at risk of being overlooked when accommodated on medical wards instead of their designated surgical units. The dependence on clinical teams for identification in preoperative clinics and cardiothoracic wards suggests that expanding research teams and implementing systematic daily screening protocols could improve recruitment rates. Strategies such as liaising with bed managers to identify all potentially eligible admissions including interhospital transfers, developing electronic newsletters for staff explaining study relevance and eligibility criteria, and providing multiple team presentations could enhance recruitment efficiency [33]. Additionally, allowing clinical staff to experience the CCT intervention during face-to-face meetings could improve their understanding and engagement with the recruitment process [33].

The recruitment of women presented challenges, reflecting the broader issue of the underrepresentation of women in cardiovascular research [34]. While the study ultimately achieved representative female participation (24%), based on local (23%) and national (23–28%) proportions of women undergoing cardiac surgery [35], this required targeted recruitment strategies. The first female participant was the 20th enrolled, highlighting initial recruitment difficulties, with 15 women approached before successful recruitment. Barriers to female participation included language proficiency issues (33% of approached women), surgery scheduled within 24 h (33%), and lack of interest (27%). The digital gender divide may contribute to reduced female participation in technology-based interventions [36], suggesting that providing dedicated devices with locked applications could overcome access barriers. Future studies should consider implementing targeted strategies including monetary reimbursement, flexible follow-up scheduling, onsite childcare, and social media outreach to relevant communities [34].

Adherence to the CCT demonstrated mixed results, with 31% of participants completing the required training time. While this aligns with reported adherence rate for home-based CCT interventions (25–30%) [37], it highlights the challenges inherent in sustained engagement with CCT programmes. Non-adherent participants typically showed minimal engagement from the outset, with some participants (13%) not accessing the training at all and others engaging for only 1–9 days. Factors contributing to non-adherence included postoperative fatigue, complicated recovery processes, ongoing health problems, and frustration with specific exercises. The early postoperative period is characterised by significant physical and psychological recovery demands [10], and it is important to consider whether an 8-week, 5-days-per-week programme represents a realistic and achievable burden for patients at this stage of recovery. Only 29% of participants commenced training on the planned date, suggesting that a flexible ramp-up period accommodating variable postoperative fatigue and discharge timelines may improve early adherence in a future study. Furthermore, the 8-week training period coincided with return-to-work transitions for some participants 6 weeks postoperatively, potentially affecting later-stage adherence. The intervention dose and timing may need to be reconsidered in future studies; our instinct, to be explored with the PPIE group, is that reducing the programme to 6 weeks may better accommodate patients’ recovery trajectories whilst maintaining sufficient training volume. Participants frequently cited the repetitive nature of the exercises as reducing enjoyment over time, suggesting that incorporating a greater variety of exercises could improve sustained engagement. The weekly contact schedule was well-received by participants, with many viewing it as a helpful reminder system. This temporal consideration suggests that training duration and intensity may need adjustment to accommodate recovery trajectories and the changing demands of postoperative life. Participants’ experiences appeared to fluctuate through the training period, consistent with reports from other cognitive training studies [26]. Strategies to improve adherence could include incorporating attainable yet challenging exercises, reducing frustration through comprehensive support strategies such as providing accessible technical support contact details and implementing screen-sharing software to resolve technical difficulties promptly.

The CCT was rated as acceptable by 94% of participants who completed the TFA-Q (*n* = 16 of 17); it is important to note that these findings reflect the views of intervention completers only and may not represent the experience of the broader recruited cohort (*n* = 29). No previous studies have employed theoretical frameworks to assess cognitive training acceptability in postoperative cardiac populations, with most studies relying solely on retrospective patient satisfaction questionnaires [26]. The comprehensive assessment of acceptability constructs beyond simple satisfaction measures provides valuable insights for intervention refinement [28]. Participants appreciated the structure that training provided to their daily routine and valued regular research contact, viewing both as positive aspects of study participation. However, specific feedback regarding exercise components highlighted areas for improvement. Participants found certain auditory-based exercises, particularly sound discrimination tasks, extremely difficult and frequently rated these as their least favourite components, recommending exclusion from future studies. This feedback suggests the need for careful exercise selection and potential PPIE involvement in determining the most appropriate and engaging training components.

This study has several strengths. Two dedicated PPIE advisors provided valuable insights into research acceptability, methods, and data collection approaches. Additionally, this represents the first feasibility study exploring home-based CCT in the postoperative cardiac population in the UK context. Most significantly, no previous study has employed a theoretical framework to assess CCT acceptability in postoperative cardiac patients, making the FACCT study a unique contribution to this emerging area.

Several limitations warrant acknowledgement. While the sample size was adequate for the study’s feasibility objectives, it was small and subgroup analyses were underpowered. Cognitive outcomes were assessed using the MoCA alone, which is a screening tool and is not sufficient for formal diagnosis of POCD; whilst practical for a feasibility study, future work should consider whether MoCA findings could be used to identify patients who may benefit from more extensive neuropsychological assessment, acknowledging that comprehensive batteries are time-consuming and require specialist administration and interpretation [3]. The study protocol’s 24-h consent window, whilst designed to ensure adequate time for informed consent deliberation, resulted in the exclusion of 48% of potentially eligible patients whose surgery was scheduled within 24 h of initial contact, representing a notable constraint on recruitment efficiency. The requirement for English language proficiency and access to a computer or tablet with an internet connection may have introduced selection bias, potentially limiting generalisability to more digitally engaged and linguistically homogenous populations. Language barriers affected 30% of ineligible participants, further limiting generalisability in the ethnically diverse population served by this tertiary centre. Furthermore, the study recruited from a first-time elective cardiac surgical population with a low median surgical risk profile (EuroSCORE II 1.23), which reflects the eligibility criteria; findings may not generalise to higher-risk patients, more diverse populations, or non-elective surgical settings. Finally, retention (59%) and adherence (31%) rates fell below predefined targets, though comprehensive approaches to address these challenges have been developed for future implementation.

The findings of this feasibility study provide a clear framework for the design of a future efficacy study. Based on the evidence generated, the following modifications are recommended. Earlier recruitment pathways should be implemented, including contacting patients at the point of surgical scheduling and distributing participant information sheets in advance of preoperative assessment appointments, to maximise the consent window and reduce exclusions driven by the 24-h scheduling. The intervention dose and timing should be reviewed, with consideration given to reducing the programme from 8 to 6 weeks in consultation with the PPIE group, to better accommodate the competing demands of early postoperative recovery. Adherence strategies should be enhanced incorporating greater exercise variety and more structured motivational contact. MoCA findings could be used to identify patients who may benefit from referral for further specialist neuropsychological assessment. Finally, eligibility criteria and recruitment strategies should be broadened to include more linguistically and demographically diverse populations in alignment with the National Institution for Health and Care Research Inclusion Strategy [38], including multilingual trial literature, diverse PPIE representation, and culturally sensitive recruitment approaches.

In conclusion, this feasibility study demonstrates that recruitment of cardiac surgical patients to a home-based CCT programme is achievable, with recruitment targets met and considerable patient interest evident. However, retention and adherence rates fell below predefined targets, representing the primary challenges to be addressed before a large trial can be undertaken. The CCT was rated as acceptable by the majority of those who completed the programme, though these findings should be interpreted with caution given they reflect completers only and cannot be generalised to the broader recruited cohort. This study does not assess the clinical effectiveness of CCT, and no conclusion regarding benefit can be drawn. Progression to an efficacy study is warranted, but only with meaningful protocol modifications, including revised intervention dose and timing, enhanced adherence strategies, earlier recruitment pathways, and more inclusive eligibility criteria. The findings from this feasibility study provide a clear evidence-based framework to inform the design of a future randomised controlled trial.

## Supplementary Information

Supplementary Material 1. Supplementary Figure S1: Training activity of participants. Supplementary Figure S2: Training levels completed. Supplementary Figure S3: Responses to individual TFA-Q items. Supplementary Table S1: BrainHQ customised intervention schedule. Supplementary Table S2: Baseline characteristics (full). Supplementary Table S3: Comparison of participants who completed the research protocol to those who did not: Mann–Whitney U. Supplementary Table S4: Comparison of participants who completed the research protocol to those who did not: independent sample t-test. Supplementary Table S5: Comparison of participants who completed the research protocol to those who did not: chi-square test of independence. Supplementary Table S6: Comparison of participants who met the minimum target for minutes of training for those who did not: Mann–Whitney U. Supplementary Table S7: Comparison of participants who met the minimum target for minutes of training to those who did not.

## Data Availability

The data underlying this article are available in the article and in its online supplementary material.

## Abbreviations

FACCT: Feasibility and Acceptability of home-based Computerised Cognitive Training
MoCA: Montreal Cognitive Assessment
PPIE: Patient and Public Involvement and Engagement
RCT: Randomised controlled trial
TFA: Theoretical Framework of Acceptability
TFA-Q: Theoretical Framework of Acceptability Questionnaire
