# Supplementary material for: Feasibility and acceptability of home-based computerised cognitive training after cardiac surgery
Source: Pilot Feasibility Stud. 2026 Jul 16;12:144. doi: 10.1186/s40814-026-01882-y (PMC13651069; doi:10.1186/s40814-026-01882-y)
Supplement: Supplementary file 1 [file 40814_2026_1882_MOESM1_ESM.docx]

**FACCT Study Supplementary Materials**

**CONSORT 2010 checklist of information to include when reporting a pilot or feasibility trial**

**Supplementary Figures**

Supplementary Figure 1 Training activity of participants

Supplementary Figure 2 Training levels completed

Supplementary Figure 3 Responses to individual TFA-Q items

**Supplementary Tables**

Supplementary Table 1 BrainHQ customised intervention schedule

Supplementary Table 2 Baseline characteristics (full)

Supplementary Table 3 Comparison of participants who completed the research protocol to those who did not: Mann-Whitney U

Supplementary Table 4 Comparison of participants who completed the research protocol to those who did not: independent sample t-test

Supplementary Table 5 Comparison of participants who completed the research protocol to those who did not: chi-square test of independence

Supplementary Table 6 Comparison of participants who met the minimum target for minutes of training for those who did not: Mann-Whitney U

Supplementary Table 7 Comparison of participants who met the minimum target for minutes of training to those who did not

CONSORT 2010 checklist of information to include when reporting a pilot or feasibility trial*

| Section/Topic | Item No | Checklist item | Reported on page No |
| --- | --- | --- | --- |
| Title and abstract | | | |
|  | 1a | Identification as a pilot or feasibility randomised trial in the title | Title page and page 1 of manuscript |
|  | 1b | Structured summary of pilot trial design, methods, results, and conclusions (for specific guidance see CONSORT abstract extension for pilot trials) | Title page, pages 2-3 |
| Introduction | | | |
| Background and objectives | 2a | Scientific background and explanation of rationale for future definitive trial, and reasons for randomised pilot trial | 4-5 |
|  | 2b | Specific objectives or research questions for pilot trial | 5 |
| Methods | | | |
| Trial design | 3a | Description of pilot trial design (such as parallel, factorial) including allocation ratio | 5 |
|  | 3b | Important changes to methods after pilot trial commencement (such as eligibility criteria), with reasons | N/A |
| Participants | 4a | Eligibility criteria for participants | 5 |
|  | 4b | Settings and locations where the data were collected | 5 |
|  | 4c | How participants were identified and consented | 7-8 |
| Interventions | 5 | The interventions for each group with sufficient details to allow replication, including how and when they were actually administered | 2-5 and sup-plementary table 1 |
| Outcomes | 6a | Completely defined prespecified assessments or measurements to address each pilot trial objective specified in 2b, including how and when they were assessed | 7-8 |
|  | 6b | Any changes to pilot trial assessments or measurements after the pilot trial commenced, with reasons | N/A |
|  | 6c | If applicable, prespecified criteria used to judge whether, or how, to proceed with future definitive trial | 10 |
| Sample size | 7a | Rationale for numbers in the pilot trial | 6 |
|  | 7b | When applicable, explanation of any interim analyses and stopping guidelines | N/A |
| Randomisation: |  |  |  |
| Sequence  generation | 8a | Method used to generate the random allocation sequence | N/A |
|  | 8b | Type of randomisation(s); details of any restriction (such as blocking and block size) | N/A |
| Allocation  concealment  mechanism | 9 | Mechanism used to implement the random allocation sequence (such as sequentially numbered containers), describing any steps taken to conceal the sequence until interventions were assigned | N/A |
| Implementation | 10 | Who generated the random allocation sequence, who enrolled participants, and who assigned participants to interventions | N/A |
| Blinding | 11a | If done, who was blinded after assignment to interventions (for example, participants, care providers, those assessing outcomes) and how | N/A |
|  | 11b | If relevant, description of the similarity of interventions | N/A |
| Statistical methods | 12 | Methods used to address each pilot trial objective whether qualitative or quantitative | 9-10 |
| Results | | | |
| Participant flow (a diagram is strongly recommended) | 13a | For each group, the numbers of participants who were approached and/or assessed for eligibility, randomly assigned, received intended treatment, and were assessed for each objective | 11 and figure 2 |
|  | 13b | For each group, losses and exclusions after randomisation, together with reasons | 11, No randomisation |
| Recruitment | 14a | Dates defining the periods of recruitment and follow-up | 11 |
|  | 14b | Why the pilot trial ended or was stopped | N/A |
| Baseline data | 15 | A table showing baseline demographic and clinical characteristics for each group | Table 1 |
| Numbers analysed | 16 | For each objective, number of participants (denominator) included in each analysis. If relevant, these numbers  should be by randomised group | 11-16, figure 2 |
| Outcomes and estimation | 17 | For each objective, results including expressions of uncertainty (such as 95% confidence interval) for any  estimates. If relevant, these results should be by randomised group | 11-16, sup-plementary tables 3-7 |
| Ancillary analyses | 18 | Results of any other analyses performed that could be used to inform the future definitive trial | 11-16, sup-plementary tables 3-7 |
| Harms | 19 | All important harms or unintended effects in each group (for specific guidance see CONSORT for harms) | N/A |
|  | 19a | If relevant, other important unintended consequences | N/A |
| Discussion | | | |
| Limitations | 20 | Pilot trial limitations, addressing sources of potential bias and remaining uncertainty about feasibility | 20-21 |
| Generalisability | 21 | Generalisability (applicability) of pilot trial methods and findings to future definitive trial and other studies | 20-21 |
| Interpretation | 22 | Interpretation consistent with pilot trial objectives and findings, balancing potential benefits and harms, and  considering other relevant evidence | 16-21 |
|  | 22a | Implications for progression from pilot to future definitive trial, including any proposed amendments | 16-21 |
| Other information | | |  |
| Registration | 23 | Registration number for pilot trial and name of trial registry | Title page |
| Protocol | 24 | Where the pilot trial protocol can be accessed, if available | N/A |
| Funding | 25 | Sources of funding and other support (such as supply of drugs), role of funders | 22 |
|  | 26 | Ethical approval or approval by research review committee, confirmed with reference number | 5 |

Citation: Eldridge SM, Chan CL, Campbell MJ, Bond CM, Hopewell S, Thabane L, et al. CONSORT 2010 statement: extension to randomised pilot and feasibility trials. BMJ. 2016;355. This is an Open Access article distributed in accordance with the terms of the Creative Commons Attribution (CC BY 3.0) license (<http://creativecommons.org/licenses/by/3.0/>), which permits others to distribute, remix, adapt and build upon this work, for commercial use, provided the original work is properly cited.

*We strongly recommend reading this statement in conjunction with the CONSORT 2010, extension to randomised pilot and feasibility trials, Explanation and Elaboration for important clarifications on all the items. If relevant, we also recommend reading CONSORT extensions for cluster randomised trials, non-inferiority and equivalence trials, non-pharmacological treatments, herbal interventions, and pragmatic trials. Additional extensions are forthcoming: for those and for up-to-date references relevant to this checklist, see [www.consort-statement.org](http://www.consort-statement.org).

target

Minutes

Frequency

target

Number of days

Frequency

**Supplementary Figure 1 Training activity of participants (n=28^a^)**

*Note. ^a^* The participant who was discontinued (intraoperative complications) has been excluded as they never had the opportunity to access the CCT.

Levels completed

|  | Completed programme and follow-up | Lost to follow-up |
| --- | --- | --- |
| 0 | 0 | 3 |
| 1-40 | 0 | 7 |
| 41-80 | 1 | 1 |
| 81-120 | 2 |  |
| 121-160 | 4 |  |
| 161-200 | 1 |  |
| 201-240 | 0 |  |
| 241-280 | 1 |  |
| 281-320 | 1 |  |
| 320+ | 7 |  |

**Supplementary Figure 2 Training levels completed (n=28^a^)**

*Note. ^a^* The participant who was discontinued (intraoperative complications) has been excluded as they never had the opportunity to access the CCT.

Supplementary Figure 3 Responses to individual TFA-Q items (n=17)

Supplementary Table 1 BrainHQ customised intervention schedule

| **Exercise** | **Domain targeted** | **What the participant has to do** | **How the exercise adapts to the participants performance** | **How the participant is scored** |
| --- | --- | --- | --- | --- |
| **Memory exercises** | | | | |
| **Hear, hear** | Auditory memory, attention | Remember a target tone and find the matching tones as they appear. | The sounds change from beeps (a single frequency) to sweeps (a set of frequencies), the beeps and sweeps become shorter, the beeps and sweeps become closer together. | The score relates to the level of similarity in the sounds heard. The higher the level, the more similar the sounds. Correct answers lead to a progression in levels. A higher score is better. |
| **To-do list training** | Working memory | Listen to a set of instructions, remember them, and follow them in order. | Items to gather get more similar. | The score is the number of instructions remembered. As participants improve, they must remember more instructions. Correct answers lead to a progression in levels. A higher score is better. |
| **Attention exercises** | | | | |
| **Divided attention** | Attention | Watch 2 shapes as they flash on the screen and decide whether they meet certain criteria. | The categories change from colour to shape to fill interior.  The categories double up. | As the participant improves, the images flash by more quickly. This exercise is measured in milliseconds (ms). A lower score is better. |
| **Target tracker** | Attention | Track target objects as they move around the screen. | Objects travel more quickly, objects travel over a larger area, objects travel for longer, contrast decreases. | The score achieved is the number of objects the participant has been able to track. A higher score is better. |
| **Processing speed exercises** | | | | |
| **Double decision** | Useful field of view, visual processing speed | Choose which car you saw at the centre of the screen and locate where the Route 66 sign appeared in the periphery. | Distractors are added, distance from the centre increases, cars get more similar, backgrounds get more complex. | This exercise is scored in milliseconds. As the user improves, the cars and road signs flash for fewer milliseconds, giving a lower (better) score. |
| **Eye for detail** | Visual processing speed, visual working memory | Watch as three or five images briefly flash in different positions on screen. Of the pictures, some match precisely, while others are similar but not the same. Participants identify where the identical images appeared. | The images change, becoming more similar. The images are shown over a larger area on screen. The number of images gradually increases from 3 to 5. | This exercise is scored in milliseconds. As the user improves, the images flash on screen for fewer milliseconds. |

Supplementary Table 2 Participant Characteristics (full)

| **Demographic and risk factor variables** | n=29 |
| --- | --- |
| Sex  Male, n(%)  Female, n(%) | 22 (76)  7 (24) |
| *Age, (years) median (IQR)* | 63.0 (13.00) |
| Ethnicity  White, n(%)  Asian, (India, Bangladesh) n(%)  Black, n(%)  Other, n(%) | 21 (73)  4 (14)  3 (10)  1 (3) |
| Educational attainment  Postgraduate, n(%)  Bachelor’s degree, n(%)  High school / secondary, n(%)  Technical/vocational, n(%) | 7 (24)  5 (17)  8 (28)  9 (31) |
| Employment status  Employed full-time, n(%)  Employed part-time, n(%)  Retired, n(%)  Unemployed, n(%) | 13 (45)  3 (10)  11 (38)  2 (7) |
| Smoking status  Never, n(%)  Ex-smoker, n(%)  Current, n(%) | 19 (66)  9 (31)  1 (3) |
| Previous percutaneous coronary intervention  No, n(%)  > 24 hours previous admission, n(%) | 23 (82)  5 (18) |
| Preoperative heart rhythm  Sinus rhythm, n(%) | 29 (100) |
| Peripheral vascular disease (extracardiac arteriopathy)  No, n(%)  Yes, n(%) | (97)  (3) |
| EuroSCORE II, median (IQR) | 1.23 (1.20) |
| Baseline MoCA, median (IQR) | 27 (2.80) |
| **Operative variables** | |
| Type of surgery  CABG, n(%)  Valve, n(%)  Major aortic, n(%)  Other, n(%) | 11 (38)  5 (17)  11 (38)  2 (7) |
| Duration of surgery (minutes), median (IQR) | 264.0 (90.30) |
| Bypass time (minutes), mean (SD) | 104.8 (58.72) |
| Aortic cross-clamp time (minutes), mean (SD) | 86.5 (51.67) |
| **Immediate postoperative variables** | |
| Return to theatre  Yes, n(%) | 0 (0) |
| Deep sternal wound infection  Yes, n(%) | 0 (0) |
| New postoperative neurological dysfunction  Yes, n(%)  Permanent stroke, n(%) | 1 (3)  1 (3) |
| ICU length of stay (days), median (IQR) | 4.1 (2.87) |
| Postoperative length of stay (days), median (IQR) | 8.0 (7.50) |
| Total length of stay (days), median (IQR) | 10.5 (8.00) |

Supplementary Table 3 Comparison of participants who completed the protocol to those who did not: Mann-Whitney U

|  | Completion of protocol | | | |  |  |
| --- | --- | --- | --- | --- | --- | --- |
|  | Completed  protocol  (*n*=17) | | Did not complete protocol  (*n*=12) | |  |  |
| tableVariable | *Med* | *Mean Rank* | *Med* | *Mean Rank* | *Z* | *P* |
| Duration of surgery (minutes) | 215.00 | 10.47 | 287.00 | 21.42 | -3.42 | < .001 |
| ICU length of stay (days)^b^ | 4.18 | 14.71 | 3.79 | 15.42 | -.22 | .845 |
| Age | 64.00 | 16.53 | 59.00 | 12.83 | -1.15 | .263 |
| Baseline MoCA score | 27.00 | 15.79 | 27.00 | 13.88 | -.61 | .556 |
| Postoperative length of stay (days) ^c^ | 7.00 | 13.41 | 9.00 | 17.25 | -1.20 | .245 |
| Total length of stay (days) | 10.00 | 14.06 | 13.00 | 16.33 | -.71 | .499 |
| EuroSCORE II | .87 | 13.00 | 1.72 | 17.83 | -1.51 | .140 |

*Note.* ^a^ Mann-Whitney *U* ^b^ ICU length of stay was measured from ICU admission to ICU discharge, and in days with 2 decimal places. ^c^ Postoperative length of stay was measured from the time of leaving the operating theatre to hospital discharge or death, in calendar days. *Abbreviation*: Med – median.

Supplementary Table 4 Comparison of participants who completed the research protocol to those who did not: independent sample t-test

|  | Completion of protocol | | | |  |  |  |
| --- | --- | --- | --- | --- | --- | --- | --- |
|  | Completed protocol  (*n* = 17) | | Did not complete protocol  (*n* = 12) | |  |  |  |
| Variable | *M* | *SD* | *M* | *SD* | *t* | *p* | *Cohen’s d* |
| Aortic cross-clamp time (minutes) | 73.24 | 51.39 | 106.91 | 47.19 | -1.75 | .092 | .68 |
| Bypass time (minutes) | 93.12 | 60.82 | 121.25 | 53.77 | -1.29 | .210 | .49 |

*Note*. Abbreviations: M – mean; SD – standard deviation

Supplementary Table 5 Comparison of participants who completed the research protocol to those who did not^a^

|  |  | Completed protocol  n (% ^b^) | Did not complete protocol  n (% ^b^) | Total  n (% ^b^) | *χ^2^* | p | Cramer’s V |
| --- | --- | --- | --- | --- | --- | --- | --- |
| Gender | Male | 14 (82) | 8 (67) | 22 (76) | .28 | .403 | .181 |
|  | Female | 3 (18) | 4 (33) | 7 (24) |  |  |  |
| Ethnicity | White | 13 (76) | 8 (67) | 21 (73) | 2.74 | .433 | .308 |
|  | Asian (India, Bangladesh) | 1 (6) | 3 (25) | 4 (14) |  |  |  |
|  | Black | 2 (12) | 1 (8) | 3 (10) |  |  |  |
|  | Other | 1 (6) | 0 (0) | 1 (3) |  |  |  |
| Employment status | Full time | 8 (41) | 5 (42) | 13 (45) | 1.01 | .798 | .187 |
|  | Part time | 1 (6) | 2 (17) | 3 (10) |  |  |  |
|  | Retired | 7 (41) | 4 (33) | 11 (38) |  |  |  |
|  | Unemployed | 1 (6) | 1 (8) | 2 (7) |  |  |  |
| Educational attainment | Postgraduate | 3 (17) | 4 (33) | 7 (24) | 2.15 | .543 | .272 |
|  | Bachelor’s degree | 4 (24) | 1 (9) | 5 (17) |  |  |  |
|  | High school / secondary | 4 (24) | 4 (33) | 8 (28) |  |  |  |
|  | Technical / vocational | 6 (35) | 3 (25) | 9 (31) |  |  |  |
| New postoperative neurological dysfunction | No | 17 (100) | 11 (92) | 28 (97) | .03 | .414 | .225 |
|  | Permanent stroke | 0 (0) | 1 (8) | 1 (3) |  |  |  |
| Smoking status | Never | 10 (59) | 9 (75) | 19 (66) | 1.23 | .541 | .206 |
|  | Ex-smoker | 6 (35) | 3 (35) | 9 (31) |  |  |  |
|  | Current | 1 (6) | 0 (0) | 1 (3) |  |  |  |
| Hypertension | No | 4 (23) | 3 (25) | 7 (24) | .73 | .694 | .159 |
|  | Treated | 12 (71) | 9 (75) | 21 (73) |  |  |  |
|  | Unknown | 1 (6) | 0 (0) | 1 (3) |  |  |  |
| Diabetes | No | 11 (66) | 7 (59) | 18 (62) | 1.06 | .787 | .191 |
|  | Diet | 1 (6) | 1 (8) | 2 (7) |  |  |  |
|  | Oral | 4 (23) | 4 (33) | 9 (28) |  |  |  |
|  | Insulin | 1 (6) | 0 (0) | 1 (3) |  |  |  |
| Previous MI | No | 0 (0) | 1 (8) | 1 (3) | 1.47 | .480 | .225 |
|  | One | 14 (82) | 9 (75) | 23 (80) |  |  |  |
|  | Two or more | 3 (18) | 2 (17) | 5 (17) |  |  |  |
| Previous PCI | No | 14 (82) | 9 (82) | 23 (82) | .001 | 1.000 | .007 |
|  | > 24 hours, previous admission | 3 (18) | 2 (18) | 5 (18) |  |  |  |
| Angina | No angina | 10 (59) | 9 (75) | 19 (66) | 3.19 | .203 | .332 |
|  | Slight limitation | 6 (35) | 1 (8) | 7 (24) |  |  |  |
|  | Marked limitation | 1 (6) | 2 (17) | 3 (10) |  |  |  |
| Peripheral vascular disease  (extracardiac arteriopathy) | No | 17 (100) | 11 (92) | 28 (97) | 1.47 | .414 | .225 |
|  | Yes | 0 (0) | 1 (8) | 1 (3) |  |  |  |
| LVEF category | Good | 13 (77) | 9 (75) | 22 (76) | 1.58 | .454 | .233 |
|  | Moderate | 4 (23) | 2 (17) | 6 (21) |  |  |  |
|  | Poor | 0 (0) | 1 (8) | 1 (3) |  |  |  |
| Type of surgery | CABG | 8 (47) | 3 (25) | 11 (38) | 4.57 | .207 | .397 |
|  | Valve | 3 (18) | 2 (17) | 5 (17) |  |  |  |
|  | Major aortic | 4 (23) | 7 (58) | 11 (38) |  |  |  |
|  | Other | 2 (12) | 0 (0) | 2 (7) |  |  |  |

*Note.* ^a^ chi square test of independence. ^b^ % within group. *Abbreviation:* CABG - coronary artery bypass grafts

Supplementary Table 6 Comparison of participants who met the minimum target for minutes of training for those who did not

|  | Met target or not^a^ | | | |  |  |
| --- | --- | --- | --- | --- | --- | --- |
|  | Participants  met target  (*n* = 9) | | Participants did not meet the target  (*n* = 8) | |  |  |
| Variable | *Med* | *Mean Rank* | *Med* | *Mean Rank* | *Z* | *p* |
| Age | 28.00 | 9.67 | 27.00 | 8.25 | -.58 | .606 |
| Baseline MoCA score | 1.11 | 9.72 | .86 | 8.19 | -.64 | .541 |
| EuroSCORE II | 66.00 | 8.89 | 63.50 | 9.13 | -.09 | .963 |

*Note.* ^a^ Mann-Whitney U. *Abbreviation:* Med – median.

Supplementary Table 7 Comparison of participants who met the minimum target for minutes of training to those who did not^a^

|  |  | Completed protocol  n (% ^a^) | Did not complete protocol  n (%^a^) | Total  n (% ^a^) | *χ^2^* | p | Adjusted p ^b^ | Cramer’s V |
| --- | --- | --- | --- | --- | --- | --- | --- | --- |
| Gender | Male | 7 (78) | 7 (88) | 14 (82) | .28 | 1.000 |  | .127 |
|  | Female | 2 (22) | 1 (12) | 3 (12) |  |  | .125 |  |
| Employment status | Full time | 4 (44) | 4 (50) | 8 (47) | 3.24 | .356 |  | .436 |
|  | Part time | 0 (0) | 1 (12) | 1 (6) |  |  |  |  |
|  | Retired | 5 (56) | 2 (26) | 7 (41) |  |  |  |  |
|  | Unemployed | 0 (0) | 1 (12) | 1 (6) |  |  | .006 |  |
| Ethnicity | White | 8 (89) | 5 (63) | 13 (7) | 1.65 | .199 | .006 | .523 |
|  | Asian (India, Bangladesh) | 1 (11) | 0 (0) | 1 (6) |  |  |  |  |
|  | Black | 0 (0) | 2 (25) | 2 (12) |  |  |  |  |
|  | Other | 0 (0) | 1 (12) | 1 (6) |  |  |  |  |
| Educational attainment | Postgraduate | 2 (22) | 1 (12) | 3 (18) | .28 | .965 | .006 | .127 |
|  | Bachelor’s degree | 2 (22) | 2 (25) | 4 (23) |  |  |  |  |
|  | High school / secondary | 2 (22) | 2 (25) | 4 (23) |  |  |  |  |
|  | Technical / vocational | 3 (34) | 3 (38) | 6 (36) |  |  |  |  |

*Note.* ^a^ chi-square test of independence ^b^ % within group. ^c^ Bonferroni adjusted p value.
